# A Security-Awareness Virtual Machine Management Scheme Based on Chinese Wall Policy in Cloud Computing

**DOI:** 10.1155/2014/805923

**Published:** 2014-02-05

**Authors:** Si Yu, Xiaolin Gui, Jiancai Lin, Feng Tian, Jianqiang Zhao, Min Dai

**Affiliations:** ^1^School of Electronics and Information Engineering, Xi'an Jiaotong University, Xi'an 710049, China; ^2^Shaanxi Province Key Laboratory of Computer Network, Xi'an Jiaotong University, Xi'an 710049, China; ^3^Xi'an Politics Institute, Xi'an 710049, China

## Abstract

Cloud computing gets increasing attention for its capacity to leverage developers from infrastructure management tasks. However, recent works reveal that side channel attacks can lead to privacy leakage in the cloud. Enhancing isolation between users is an effective solution to eliminate the attack. In this paper, to eliminate side channel attacks, we investigate the isolation enhancement scheme from the aspect of *virtual machine* (VM) management. The security-awareness VMs management scheme (SVMS), a VMs isolation enhancement scheme to defend against side channel attacks, is proposed. First, we use the *aggressive conflict of interest relation* (ACIR) and *aggressive in ally with relation* (AIAR) to describe user constraint relations. Second, based on the Chinese wall policy, we put forward four isolation rules. Third, the VMs placement and migration algorithms are designed to enforce VMs isolation between the conflict users. Finally, based on the normal distribution, we conduct a series of experiments to evaluate SVMS. The experimental results show that SVMS is efficient in guaranteeing isolation between VMs owned by conflict users, while the resource utilization rate decreases but not by much.

## 1. Introduction

With the promotion and development of cloud computing, virtualization technology gets increasing attention by academia and industry. There is a broad consensus that virtualization technology improves the security and reliability of cloud computing. This is mainly because of the seemingly strong isolation, which prevents the guest VMs located in the same host from interfering with each other. However, such logical isolation may not be sufficient [1]. Using the *side channel attacks *(SCA), which is firstly introduced by Kocher [2], malicious users can circumvent the isolation mechanism and extract private information from other users by analyzing responses of third party shared resources [3–5].

According to whether the attacker and victim reside in the same host, we can divide SCA into interhost SCA and intrahost SCA. In the scenario of intrahost SCA, the attacker resides in the same host with victim. Using the shared resource in the host, such as data cache and instruction cache, attackers can steal the private information from the victim VMs [6–8]. In the scenario of interhost SCA, attacker and victim are not coresident. This kind of SCA is always implemented based on the network traffic, with which attackers can steal private information from the VMs located in different hosts [9, 10]. In this paper, we focus on the intrahost SCA. In the following, SCA refers in particular to the intrahost SCA.

To eliminate SCA, a variety of approaches to enhance isolation have been proposed in the cloud. We categorize them into two types, which are the approaches focused on isolating the running of VMs (see, e.g., [11–14]) and the approaches focused on isolating the shared resources (see, e.g., [15–17]). The first type of approach achieves the isolation by preventing the conflict VMs from running simultaneously. The second type of approach prohibits the sharing of shared resources. These approaches do represent major progress, while, to the best of our knowledge, they are still limited in several aspects.

- By using the first type of approach, the schedule of legitimate VMs may be affected, which may lead to VMs running failure. For example, oil-A and oil-B are two coresident VMs, and they are in conflict with each other. So, in the system with such access control mechanism, before the shutdown of oil-A, oil-B cannot be granted to start up. If the oil-A is long-time running, oil-B cannot be scheduled in a long time.
- When using the second type of approach, the monitoring system and mediation mechanism are needed, which should probe all resource requests made by each VM and make decisions on whether to authorize the requests quickly [12]. So, the software system is very sophisticated. For example, in a SELinux strict policy, there are about 30 000 policy statements. On the other hand, a prohibitively large number of operating system hooks (in the order of hundreds) are needed, which makes the MAC policies for these systems depend on details of the particular system and the enforcement across a distributed system difficult.

In this paper, we investigate the isolation issue from the perspective of VMs management, including VMs placement and VMs migration. The *security-awareness VMs management scheme* (SVMS) based on Chinese wall policy is proposed. Compared with existing isolation enhancing schemes, the main contributions of this work are listed as follows.

- We calculate the *aggressive conflict of interest relation* (ACIR) for the users according to the VMs traces. On the other hand, by introducing the *aggressive in ally with relation* (AIAR), we can restrain the placement behavior for the new users who do not have VMs traces.
- Based on the Chinese wall policy, we put forward the isolation rules. In these rules, we define whether to authorize the request of placing VMs to host. And we prove that these rules meet the simple-security property and *-security property.
- According to the isolation rules, we design the VMs placement and migration solution calculation algorithm, which places and migrates the VMs to the proper hosts to guarantee the isolation between conflict users.
- We conduct a series of simulated experiments to evaluate the efficacy of SVMS. In the experiments, we use the normal distribution to simulate the users' behavior, including the VMs traces and VMs requests, and introduce the index of isolation degree and resource utilization rate.

The remainder of this paper is organized as follows.  Section 2 reviews the related work.  Section 3 demonstrates the brief introduction to SCA in the cloud and Chinese wall security. In Section 4, we present the overview design of SVMS. The implementation details of SVMS are presented in Section 5. In Section 6, we illustrate our experiments and analyze the experimental results.  Section 7 provides the conclusions.

## 2. Related Work

### 2.1. Studies on Enhancing Isolation

Studies on enhancing isolation in the virtualized computing environment can be categorized into two types: the access control approaches and the resource isolation approaches.

*Access Control Approaches*. Sailer et al. [11] presented the *sHype* hypervisor security architecture, which enforced isolation at the granularity of a virtual machine. McCune et al. [12] introduced a *Shamon* approach for MAC enforcement across distributed systems that complete MAC reference monitoring from two software layers. Jaeger et al. [13] evaluated the ability of four policy models to express risk flow policies and examined how such policies would be enforced in VM systems to assess the possible risk of information leakage due to a combination of overt channels and covert channels. Cheng et al. [14] proposed the prioritized Chinese wall model to reduce the risk of covert flows in VM system and enforced the policy in sHype/Xen system. The above works focus on prohibiting the simultaneous running of conflict VMs by specific access control policies.

*Resource Isolation Approaches*. Raj et al. [15] proposed two resource management approaches to provide security isolation in the shared cloud infrastructure, which are cache hierarchy aware core assignment and page coloring based cache partitioning. Yasusi et al. [16] proposed two methods to achieve resource isolation among virtual networks, which was per-slice shaping and per-link policing. Jin et al. [17] proposed a cache partitioning mechanism in the cloud, which confined the L2 cache usage of each VM running on the same host by modifying the page allocation algorithm in the VMM. The above works focus on prohibiting the sharing of shared resource to provide strong isolation in the virtualized computing environment.

### 2.2. Studies on VM Placement

Speitkamp and Bichler [18] presented decision models to optimally allocate source servers to physical target servers and a heuristic to address large-scale server consolidation projects. Wang et al. [19] formulated the VM consolidation into a stochastic bin packing program and proposed an online packing algorithm. Kim et al. [20] placed virtual machines with matching algorithm into servers based on measured traffic distribution of VMs which could reduce oversubscription and increase link utilization. Breitgand and Epstein [21] improved the performance of work in [19], which abstracted oversubscription factors as overflow probability. Fang et al. [22] presented an approach, named VMPlanner, to optimize both virtual machine placement and traffic flow routing so as to turn off as many unneeded network elements as possible for power saving.

In the field of VMs placement, there are lots of outstanding achievements, and we only list some significant ones. And we can discover that the existing works focus on optimizing allocation of resources. Although they are different from our work, they provide us with a good reference. And this paper represents a significant extension to our prior work [23].

## 3. Background: SCA and the Chinese Wall Policy

In this section, we briefly introduce (1) SCA in the cloud and (2) the Chinese wall policy.

### 3.1. SCA in the Cloud

In the cloud, to maximize efficiency, multiple VMs owned by different users may be simultaneously assigned to execute on the same physical host. So, it is possible for the attackers to use SCA to penetrate the isolation between coresident VMs and extract private information.

According to the *Trusted Computer Security Evaluation Criteria* (TCSEC), a side channel attack is any attack based on information gained from the physical environment, as opposed to brute force or theoretical weaknesses [24]. For example, timing information, power consumption, or electromagnetic leaks can bring extra information. Commonly, in the cloud, SCA consists of two major steps: placement and extraction [4]. In the first step, the attackers should try to locate their VMs in the same host with the VMs of victim. According to [4], the attackers can achieve this with considerable possibility in the Amazon EC2. In the second step, the attackers first probe the usage of shared resource. Then, based on the relationship between the shared resource usage and private information, the attackers design the leakage model to extract the private information from the probed usage samples.

In the cloud, researchers have investigated how to use SCA to steal information from other users. For example, Zhang et al. [5] used the L2 cache based side channel analysis as a defensive detection tool to exploit the malicious coresident VM. Zhang et al. [25] used the L1 instruction cache as side channel to extract the decryption key from the coresidency VM. Okamura and Oyama [6] developed CCCV, which created a side channel and communicated data secretly using CPU loads between the Xen VMs. Xu et al. [7] quantified the L2 cache cross-VM side channel with the *bit rates* and assessed their ability to do harm.

### 3.2. Chinese Wall Policy

Chinese wall policy is firstly proposed by Brewer and Nash [26], which is also called BN Chinese wall policy. It has a three hierarchy architecture, including lowest level, intermediate level, and highest level. The lowest level consists of individual objects. The intermediate level consists of the company datasets which contain objects related to a single company. The highest level consists of conflict of interests which contains the datasets of companies in competition.

According to the definition of Brewer and Nash, Chinese wall policy has the following two properties [26].

- *Simple-security property*. An object O can be read if the subject has accessed a prior object O′ belonging to the same dataset or the objects conflict of interest set is new.
*-security property. Simple-security property should be fulfilled for the target O and the writer has only read data from the company dataset of O.

Since the Chinese wall policy takes advantage of the mandatory enforcement and independent choice of users, it has been applied in many fields. For example, various access control models are proposed based on Chinese wall policy, which can be implemented in the cloud effectively [27, 28]. Furthermore, Chinese wall policy is also applied in the field of delegation [29].

In this paper, we focus on the variety of BN Chinese wall policy, the aggressive Chinese wall policy which was proposed by Lin [30]. And we will use the notations presented in [30], such as ACIR and AIAR, to illustrate the constraint relations for users.

## 4. Design of SVMS

**Figure 1:**
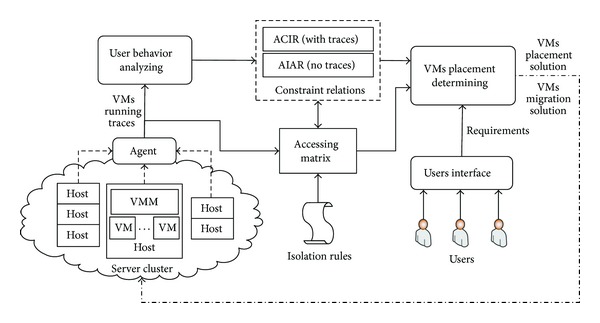
Overview of the architecture design for SVMS.

In this section, we provide a high-level overview of the architecture for SVMS. As shown in Figure 1, SVMS consists of three major components, which are *behavior sampling*, *constraint relations analyzing,* and *placement determining*.

1. *Behavior sampling.* In each host, we deploy the agent to record the behavior of VMs, thus obtaining the VMs traces.
2. *Constraint relations analyzing.* In this component, we analyze the constraint relations. For the users who have VMs traces, we calculate the ACIR. For the new users who do not have VMs traces, we calculate the AIAR. Then, combining the isolation rules and constraint relations, we can get the access matrix which records the maps between VMs and hosts and provides feedback for the dynamical update for constraint relations.
3. *Placement determining.* According to the constraint relations and access matrix, this component calculates the VMs placement solutions. On the other hand, when the ACIR of users changes, this component also provides the VMs migration solutions to guarantee the isolation between conflict users.

## 5. Implementation of SVMS

In this section, we describe the implementation details of SVMS. First, we present the ACIR computation process. Second, based on the Chinese wall policy, we put forward the isolation rules. Third, we propose the VMs placement solution and migration calculated algorithm.

### 5.1. Computing the ACIR

In the BN Chinese wall policy, conflict of interest (CIR) is an equivalence relation. However, in the scenario of this paper, CIR is a binary relation which is reflexive, symmetric, and not transitive. For example, oil-A, oil-B, and bank-C are three VMs, where oil-A and oil-B are prohibited to reside in the same host; oil-B and bank-C are also prohibited to reside in the same host. However, we cannot deduce that oil-A and bank-C are prohibited in the same host. So, based on the work of Lin [30], we use the ACIR to describe the CIR for users.

**Definition 1**

Aggressive Conflict of Interest Relation (ACIR). If user *u*_*i*_ is aggressive conflict with user *u*_*j*_, denoted as *u*_*i*_ ACIR *u*_*j*_, then the VMs owned by *u*_*i*_ are prohibited to reside in the same host with the VMs owned by *u*_*j*_, and ACIR(*u*_*i*_) is called as the ACIR set for *u*_*i*_.

According to the previous analysis, we compute the ACIR for users according to the VMs traces. To formulate the VMs traces, we define a specific time period as a placement cycle. And the placement cycle is divided into several *time domains *with the same time span. Then we define the number of active VMs in different time domains as *VMs traces* for different users.

Based on the work of Pawlak [29], we put forward the following steps to compute ACIR sets according to the VMs traces.

**Step 1**

Forming the information table (IT). IT contains three major attributes: user (*u*_*j*_), time domain (*td*_*k*_), and VMs traces value (*σ*_*l*_ equals to the support factor defined in [29]), as shown in Table 1.

**Step 2**

Computing the strength factor and certainty factor for each record in IT. For the record {id_*i*_, *u*_*j*_, td_*k*_, *σ*_*l*_}, the strength factor and certainty factor are defined in (1). Consider

(1) $$\begin{array}{c} {\text{strength}}_{i}=\frac{\sigma_{l}}{\sigma \left(\text{IT}\right)},\\ {\text{certainty}}_{i}={\text{strength}}_{i}*\frac{\sigma \left(\text{IT}\right)}{\sigma \left(u_{j}\right)}, \end{array}$$

where *σ*(IT) refers to the summary of support factor for all the records in IT; *σ*(*u*_*j*_) refers to the summary of support factor for user *u*_*j*_ in IT.

**Step 3**

Computing the active time domains. For each record in IT, if the certainty factor is larger than the threshold (e.g., 0.2), we consider that the user is active in this time domain. The denotation “*u*_*j*_ → *td*_*k*_” is used to represent that user *u*_*j*_ is active in time domain *td*_*k*_. So, for any user *u*_*j*_, the active time domain is **T**_*j*_ = {td_*k*_ | *u*_*j*_ → td_*k*_}.

**Step 4**

Forming the similarity matrix **S**{*s*_*ij*_}, where *s*_*ij*_ refers to the similarity between *u*_*j*_ and *u*_*j*_. Consider

(2) $$\begin{array}{c} s_{ij}=\{\begin{array}{cc} 0; & i=j\;\text{or}\;\left|T_{j}\right|=0\\ \frac{\left|T_{i}\cap T_{j}\right|}{\left|T_{j}\right|};\; & \text{otherwise}, \end{array} \end{array}$$

where |**T**_*j*_| refers to the cardinality of **T**_*j*_.

**Step 5**

Obtaining the ACIR sets. If *s*_*ij*_ is larger than 0, then *u*_*i*_ ACIR *u*_*j*_, which means *u*_*j*_∈ ACIR(*u*_*i*_). Finally, we can get the ACIR sets for the users who have VMs traces.

### 5.2. The Isolation Rules and AIAR

For the users who have the VMs traces, using the previous steps, we can easily obtain their ACIR sets. However, for the new users who do not have VMs traces, we cannot compute the ACIR sets for them by using these steps. To solve this problem, we introduce the AIAR. For the new users, their VMs could only be placed to the hosts which are occupied by the users in their AIAR sets. In this section, we first define AIAR. Then, we propose the isolation rules.

**Definition 2**

Aggressive In Ally with Relation (AIAR). If user *u*_*i*_ is aggressive in ally with *u*_*j*_, denoted as *u*_*i*_ AIAR *u*_*j*_, then the VMs owned by *u*_*i*_ are permitted to reside in the same host with the VMs owned by *u*_*j*_, and AIAR(*u*_*i*_) is called the AIAR set for *u*_*i*_.

AIAR is an equivalence relation which is reflexive, symmetric, and transitive. And we will present how to compute AIAR in the isolation rules. Consider

(3) $$\begin{array}{c} a_{ij}=\{\begin{array}{cc} -1, & \text{against}\\ 0, & \text{neutral}\\ 1, & \text{favorable}. \end{array} \end{array}$$

In this paper, we define the access matrix **A**{*a*_*ij*_} as a two-dimensional matrix: **A** : **H** × **U** → {−1,0, 1}. The notations “1”, “0”, and “−1” mean that the VM is favorable, neutral, and against to be placed to the host, respectively, as shown in (3). If the VM placement request to *h*_*i*_ from *u*_*j*_(*R*(*i*, *j*)) is rejected, then the entry *a*_*ij*_ is assigned to “−1”; if *R*(*i*, *j*) is granted, then *a*_*ij*_ is assigned to “1”; and if *R*(*i*, *j*) is not determined, than *a*_*ij*_ is assigned to “0”.

Based on the access matrix, we put forward the isolation rules.

**Rule 1**

Initially, for all *i* and *j*, *a*_*ij*_ = 0.

Initially, each request *R*(*i*, *j*) is neutral. In other words, any VMs are neutral to be placed to any hosts initially.

**Rule 2**

*a*_*ij*_ = 0⇒*R*(*i*, *j*) is granted ∧ *a*_*ij*_ = 1∧∀*l* ≠ *j*, *u*_*l*_ ∈ *AIAR*(*u*_*j*_), *a*_*il*_ = 1∧∀*l* ≠ *j*, *u*_*l*_∈ACIR(*u*_*j*_), *a*_*il*_ = −1∧ update AIAR(*u*_*j*_) according to Rule 5.

In this rule, we define the placement behavior when *u*_*j*_ is neutral to place VMs to *h*_*i*_. When the access permission is neutral, we authorize that user *u*_*j*_ can place VMs to the host. Moreover, the users in AIAR(*u*_*j*_) are also authorized to place VMs to the host. However, the users in ACIR(*u*_*j*_) are not allowed to place VMs to the host.

This rule applies especially for new users, whose ACIR and AIAR are empty initially. In this scenario, the first access request sent by new users is always granted. Then, the AIAR is initialed, with which the behavior of new users is restrained in the placement cycle.

**Rule 3**

*a*_*ij*_ = 1⇒*R*(*i*, *j*) is granted ∧ ∀*l* ≠ *j*, *u*_*l*_ ∈ ACIR(*u*_*j*_), *a*_*il*_ = −1 ∧ ∀*l* ≠ *j*, *u*_*l*_ ∈ AIAR(*u*_*j*_), *a*_*il*_ = 1∧ update AIAR(*u*_*j*_) according to Rule 5.

The placement behavior when *u*_*j*_ are favorable to place his VMs to *h*_*i*_ is defined in this rule. If *u*_*j*_ is granted to access *h*_*i*_ or has accessed *h*_*i*_, we authorize this request and grant the users in AIAR(*u*_*j*_) to access *h*_*i*_. However, the users in ACIR(*u*_*j*_) are rejected to access this host.

**Rule 4**

*a*_*ij*_ = −1⇒*R*(*i*, *j*) is denied.

If *u*_*j*_ is against to access *h*_*i*_, we denied his requests to place VMs to *h*_*i*_.

**Rule 5**

*R*(*i*, *j*) is granted ∧*AICR*(*u*_*j*_) = = NULL ⇒∀*l* ≠ *j*,*a*_*il*_ = 1, *AIAR*(*u*_*j*_) = {*l*} ∪  *AIAR*(*u*_*l*_) ∪ *AIAR*(*u*_*j*_).

In this rule, we update AIAR for new users dynamically. If *u*_*j*_ is authorized to access *h*_*i*_, then AIAR(*u*_*j*_) should be expanded by adding two kinds of users, including (1) the user *u*_*l*_ who is granted to access *h*_*i*_ and (2) the users in AIAR(*u*_*l*_).

We put forward the above five rules based on Chinese wall policy. According to our scenario, we use the *access* permission to substitute for *read* and *write *permission. If the object has the access permission, then he has the read and write permission. And we can prove that the rules meet the simple-security property and *-security property, which will be detailed in the appendix.

### 5.3. The VMs Placement and Migration Solutions

In this section, based on the constraint relations and isolation rules, we propose the VMs placement solution calculated algorithm to get the placement solutions. And we propose the VMs migration solution calculated algorithm to obtain the migration solutions when changing the ACIR of users.

#### 5.3.1. The VMs Placement Solution Calculated Algorithm

The basic idea of this algorithm is as follows. First, according to user type (new user or not) and the isolation rules, we obtain the candidate hosts which the user can access. Then, we determine whether the available space of candidate hosts is able to meet the needs of user. If the available space is not enough, we add new hosts to the candidate hosts. Finally, we place the VMs to the candidate hosts and update the access matrix and constraint relations (AIAR for new users).

The detailed processes of this algorithm are shown in Algorithm 1.

Regarding the input parameters for this algorithm, id refers to the user identity; ur refers to the counts of requested VMs; *A* refers to the access matrix with *m* rows and *n* columns; acir refers to the ACIR set for all the users in the system. Finally, we output the updated access matrix.

In this algorithm, we introduce the following functions. update() adds a new column to the access matrix when user new is joined in. firstFitHost() is designed for the new user, which gets the first available host and returns the identity of this host. getAiar() initials the AIAR set for new user. getCandHost() returns the available hosts which the user has the access permission to according to his constraint relations. There are two calling mode for getCandHost(). The first mode is designed for the new user, which should input the AIAR set and access matrix. The second mode is designed for the user who has VMs traces, which should input id, ACIR set, and access matrix. addNewHosts() is used to add new hosts to the candidate hosts when the space cannot meet the user request. getSolution() deploys the VMs to the candidate hosts.

#### 5.3.2. The VMs Migration Solution Calculated Algorithm

In the scenario of multiplacement cycles, ACIR changes with the change of VMs traces. So, the VMs owned by conflict users may be placed in the same host when changing the ACIR. To solve this problem, we use VMs migration to guarantee the isolation between conflict users.

The detailed processes for this algorithm are shown in Algorithm 2.

In this algorithm, we first look for the host where the VMs owned by conflict users reside. Then, we calculate the minimum number of VMs which should be migrated to remove the conflict. Finally, we obtain the candidate destination hosts for the migrated VMs.

In this algorithm, *h*[*i*] records the identity of users whose VMs locate in host *h*_*i*_, where *u* refers to the user identity. coVMs[] records the users whose VMs are colocated with user *u*, and acirU[] records the ACIR of *u*. confVM[] refers to the union of coVMs[] and acirU[], which is calculated by sameElem(). *nU* is an integer, which refers to the number of VMs of *u* located in *h*_*i*_; nConf is a structure, which records *nU* for each user in confVM[] and the user identity. The above two variables can be obtained by num().

## 6. Experiments

The overall evaluation of SVMS comprised of (1) experiments to evaluate the feasibility of ACIR set calculation and (2) experiments to evaluate the performance of SVMS with the index of isolation degree and resource utilization.

### 6.1. Feasibility of ACIR Calculation

In this experiment, we mainly focus on evaluating the feasibility of calculating ACIR for different users.

*Experimental Data Preparation.* In the simulated experiment, we set one day as a placement cycle which was divided into 8 time domains with the same time span. 80 users were simulated, and they were divided into 8 groups (group_*i*_, *i* = 1~8). Each group consisted of 10 users.

The normal distribution *X* ~ *N*(*μ*, *σ*) was used to simulate the VMs traces for different users, where the cumulative probability in the range of [*x* − 0.5, *x* + 0.5] was used as the probability that VMs were active in time domain td_*i*_. By converting the normal distribution to standard normal distribution *Y* = (*X* − *μ*)/*σ*, we could calculate the probability as (4). Consider

(4) $$\begin{array}{c} p\left(\text{t}{\text{d}}_{i}\right)=\Phi \left[\frac{\;\text{t}{\text{d}}_{i}+0.5-\mu \;}{\sigma }\right]-\Phi \left[\;\frac{\text{t}{\text{d}}_{i}-0.5-\mu }{\sigma }\right]. \end{array}$$

**Figure 2:**
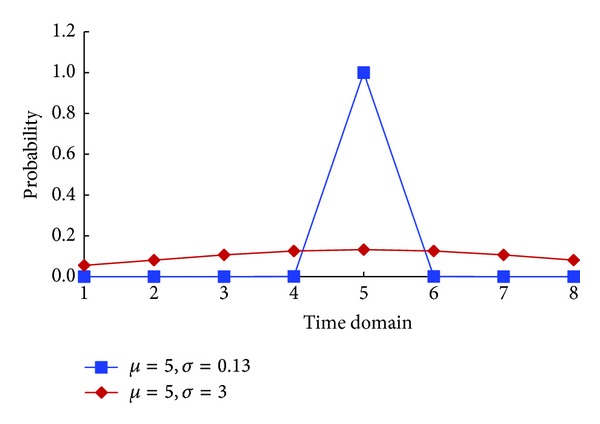
The cumulative probabilities for different time domains.

To obtain the probabilities, two key parameters should be set, which were the expectation *μ* and standard deviation *σ*. For the users in group_*i*_, we set the expectation as td_*i*_. The standard deviation was simulated randomly generated from a specific range [*σ*_min⁡_, *σ*_max⁡_]. Using *σ*_max⁡_, we could state that the probability for each group was similar with each other. Using *σ*_min⁡_, we could state that the probability for the specific group was close to 1 and the probabilities for the other groups were close to 0. According to the 3*σ* principle, 3*σ*accounted for about 99.73%. So when 3*σ*-(−3*σ*) equaled to 1, we could insure that about 99.73% of the total VMs would be active in one time domain. In this case, *σ* equaled to 0.13. According to the theoretical calculation, we could observe that when assigning 3 to *σ*_max⁡_, the probability for each time domain was the most similar. Therefore, in the experiment, we randomly generated *σ* in the range [0.13,3]. In Figure 2, we demonstrated some significant results when *μ* was equal to 5.

We used the random integer to describe the total number of VMs (*η*_*i*_) owned by user *u*_*i*_, where the range is [1, 50]. So, the number of active VMs owned by *u*_*i*_ in time domain td_*j*_ was ⌊*η*_*i*_∗*p*(td_*j*_)⌋.

Finally, we could simulate the VMs traces for the users in one placement cycle. And the ACIR set for each user could be calculated. In the experiment, we repeated the process for 100 times. In the repeated processes, the expectation of users was constant, while the standard deviation and total number of VMs owned by users were randomly generated for each time.

To quantify the aggressive conflict relations between users, we defined the average conflict times.

**Definition 3**

Average Conflict Times (act). If user *u*_*k*_ (*u*_*k*_ ∈ *group*_*i*_) was aggressive conflict with *c*_*kj*_ users in group_*j*_, then average conflict times between group_*i*_ and group_*j*_ (act_*ij*_) are defined as (5). Consider

(5) $$\begin{array}{c} \text{ac}{\text{t}}_{ij}=\frac{1}{m}\underset{u_{k}\in \text{grou}{\text{p}}_{i}}{\sum }c_{kj}. \end{array}$$

Since the user is not aggressive conflicted with himself, so, if *i* = *j*, *m* = |*group*_*i*_ | −1; if *i* ≠ *j*, *m* = |*group*_*i*_|.

**Figure 3:**
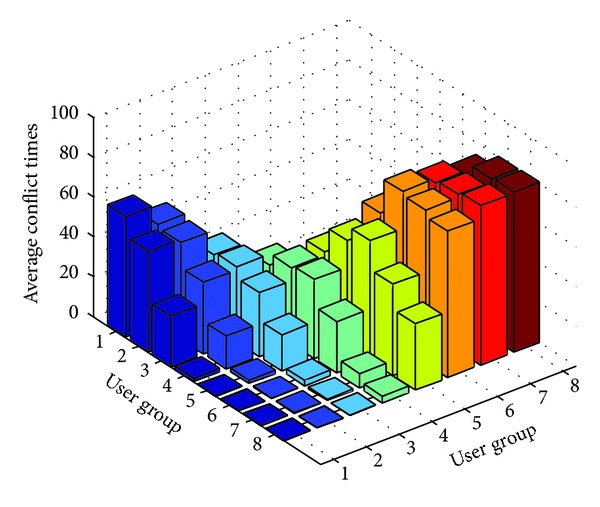
The average conflict times between different user groups.

Finally, the experimental results for average conflict times were shown in Figure 3.

Taking group_1_ as an example, from Figure 3, we could easily observe that the act_11_ was about 60; act_12_ was about 50; act_13_ was about 25; and act_1*x*_ (*x* > 3) was 0. For other groups, we could observe the similar experimental results, where the value of act_*ij*_ decreased with the increase of distance between group_*i*_ and group_*j*_. In other words, the regular in conflict times was consistent with the behavior of users (VMs traces).

*Brief Summary*. In the experiment, based on normal distribution, we generated the simulated VMs traces. Considering the diversity of users' behavior, we divided the users into different groups. The users in the same group had the same expectation, while the standard deviation was randomly generated. According to the experimental results, we could draw the following conclusions: (1) the more the similarity between VMs traces for different users, the larger the conflict probability between the users would be; and (2) according to the VMs traces, we could obtain the ACIR set for users effectively by using the conflict analysis approach.

### 6.2. Evaluating the Isolation and Resource Utilization

Through the theoretical proof we could know that our proposed isolation rules met both simple-security property and *-security property. In this section, we aimed to evaluate the possibility for the VMs owned by different users to be located in the same host.

In the experiments, we used the index of *isolation degree* and *resource utilization rate* to evaluate the performance of SVMS, compared with the existing *resource-awareness VM placement schemes* (RVMPS). A variety of RVMPS specific to different scenarios were proposed (see, e.g., [18–22]). In our experiments, we considered that the basic idea for RVMPS was to use the fewest resources to meet the VMs needs of users.

**Definition 4**

Coresidency Times (ct). If there are *c* hosts in which the VMs owned by *u*_*i*_ and *u*_*j*_ reside, then the coresidency times between *u*_*i*_ and *u*_*j*_ are *c*, where *i* ≠ *j*, which is denoted as *ct*_*ij*_ = *c*.

**Definition 5**

Isolation Degree (*ι*). For any user *u*_*i*_ and *u*_*j*_, *i≠j*, if *u*_*j*_∈ ACIR(*u*_*i*_) and ct_*ij*_ = *c*; then the isolation degree for the system is defined as (6)

(6) $$\begin{array}{c} \iota =\frac{1}{1+0.5*\sum_{i}^{}\text{c}{\text{t}}_{ij}}. \end{array}$$

According to Definition 5, if VMs owned by conflict users were not placed in the same host, which meant that ∑ct_*ij*_ = 0, then the isolation degree was 1. In this case, the efficacy of isolation was optimal. On the contrary, a larger value of ct for conflict users referred to a smaller value of *ι*, which meant that the isolation of the placement solution was inefficient.

**Definition 6**

Resource Utilization Rate (rur). The maximum number of VMs which simultaneously run in host *h*_*k*_ was *a*_*k*_, and the real *number* of VMs run in this host was *b*_*k*_, then the resource utilization rate for this host was *b*_*k*_/*a*_*k*_. For the system, the resource utilization rate was defined as (7). Consider

(7) $$\begin{array}{c} \text{rur}=\frac{\sum_{k}^{}b_{k}}{\sum_{k}^{}a_{k}}. \end{array}$$

In this section, we used the same approach to generate the VMs requests for different users, where the number of requested VMs for user *u*_*i*_ in time domain *td*_*j*_ was *r*_*ij*_ = ⌊*p*(td_*j*_)∗*η*_*i*_⌋. And we considered the case of one placement cycle and the case of multiplacement cycles.

#### 6.2.1. The Case of One Placement Cycle

In the case of one placement cycle, the VMs traces would not be recounted. So, ACIR for users would not change. In this case, we considered two scenarios, including (1) no new user joined in and (2) one new user joined in, to evaluate the performance of SVMS and the impact of new user. Using the repeated tests, we obtained the isolation degree and resource utilization rate.

**Figure 4:**
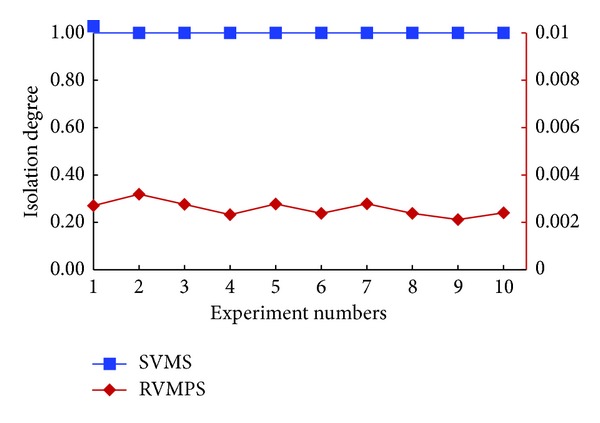
Isolation degrees when no new users joined in.

**Figure 5:**
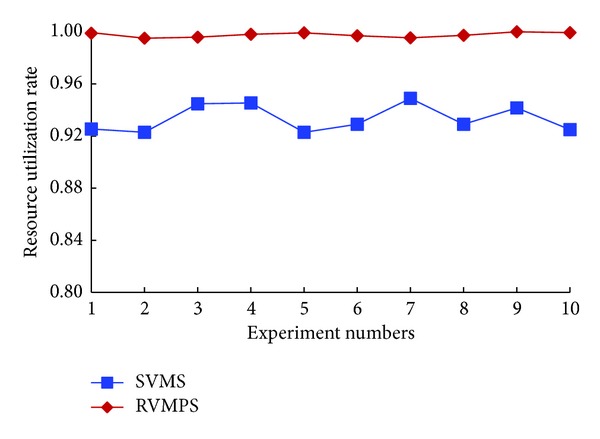
Resource utilizations when no new users joined in.

*(1)    The Scenario of No New Users Joined In*. In the scenario of no new users joined in, using the above approaches to generate the test data, we compared SVMS with RVMPS; the experimental results are shown in Figures 4 and 5.

In Figure 4, we demonstrated the experimental results of isolation degree in the scenario of no new users joined in. The black vertical on the left side referred to the coordinates for the “SVMS” curve. And the red vertical on the right side referred to the coordinates for the “RVMPS” curve. From this figure, we could easily observe that the isolation degree was kept as 1 in the repeated 10 tests, which meant that SVMS could guarantee that VMs owned by conflict users would not be placed to the same host. However, the isolation degree of RVMPS was very small, which was about 0.002 to 0.004 in the repeated tests. The small value of isolation degree meant that coresidency times for conflict users were about 500 to 1000. Generally, coresidency times for conflict users were related with the number of VMs owned by the conflict users and scheduling order of users. Due to the limited space, we will not investigate these factors here. Overall, in the same experimental settings, compared with SVMS and RVMPS, we could conclude that the VMs owned by conflict users would be placed to the same host with high probability when the conflict relations were not considered, which could introduce the vulnerability of side channel attacks.

In Figure 5, we demonstrated the resource utilization rate for SVMS and RVMPS. From this figure, we could observe that rur was all close to 100% by using RVMPS, which meant that all the hosts could run at full load. While by using SVMS, rur was less than the resource utilization rate of RVMPS, which was about 92% to 96%. The experimental results indicated that SVMS made a trade-off between the isolation and resource utilization. In other words, by using SVMS, we guaranteed the isolation between conflict users by using more physical resources.

*(2) The Scenario of New User Joined In*. In this experiment, we considered new users joined in and evaluated the impact of new users. Implicitly, we considered only one new user, since the placement behavior of new users was determined by their AIAR sets and had nothing to do with ACIR sets of other users. In this scenario, for the new user, we did not need to generate his VMs traces, and we used *r*_*nj*_ = ⌊*p*(td_*j*_)∗*η*_*i*_⌋ to refer to the number of requested VMs in time domain *td*_*j*_. For other users, we used the same approach to generate the test data.

**Figure 6:**
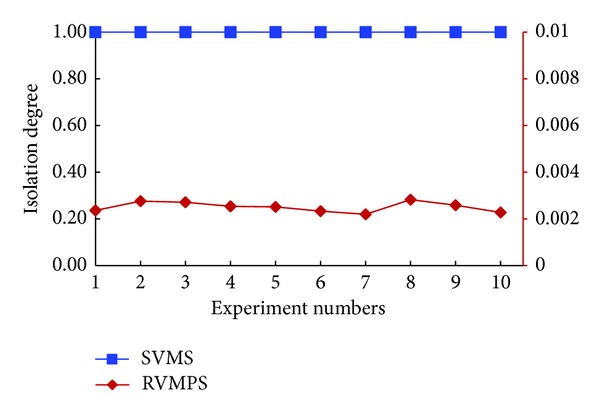
Isolation degrees when one new user joined in.

**Figure 7:**
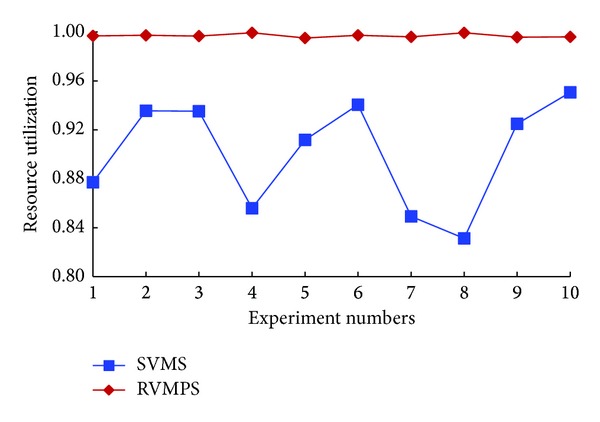
Resource utilizations when no new users joined in.

Using the same experimental steps, we could obtain the experimental results for isolation degree and resource utilization rate, as shown in Figures 6 and 7.

In Figure 6, the black vertical on the left side referred to the coordinates for the “SVMS” curve. And the red vertical on the right side referred to the coordinates for the “RVMPS” curve. As shown in Figure 6, the experimental results for isolation degree were similar with the experimental results shown in Figure 4, regardless of SVMS or RVMPS, where id for SVMS was 1 and id for RVMPS was about 0.002 to 0.004. Although only one new user was considered, we could conclude that new users had no impact on the isolation between the conflict users.

In Figure 7, we demonstrated the results of resource utilization rate when a new user joined in. Comparing the results shown in Figures 7 and 5, we could observe that the new user had no impact on rur when using RVMPS to get the placement solutions, for the rur was close to 1 with these two schemes. However, using SVMS made a difference on rur from using RVMPS. First, the value of rur was smaller. By using SVMS, the smallest rur was about 83% and the average rur was about 90%. Second, the variance of rur was larger. The difference between the largest rur and smallest rur was about 12%. There were mainly two reasons. First, in SVMS, we used the AIAR to restrain the target location of VMs owned by new users, which would reduce the range of available candidate hosts. Then we started more new hosts, resulting in more resource wastes. Second, the AIAR set was initialed and dynamically updated according to the first fit host, while the first fit host was chosen randomly, and the VMs requests of new users were also generated randomly. As a result the value of rur in repeated tests would fluctuate with the deployment situation of the first fit host and the VMs requests.

#### 6.2.2. The Case of Multiplacement Cycles

To investigate the performance of SVMS in isolation and resource utilization, we only considered one placement cycle previously. As analyzed above, in this case, AIAR of users did not change. However, the cloud is dynamic; VMs traces would change at any time, which brought about the change to the ACIR sets. When ACIR of users changed, in SVMS, we used the VMs migration to guarantee the isolation between conflict users. In this section, we mainly investigated the impact of VMs migration on system performance. And the indexes of migration ratio and resource utilization were used.

In this experiment, we used the same approaches to generate the test data for the users who had VMs traces. For the new users, we defined a random function *ranControl*()→{0,1}. *ranControl* randomly returned “0” and “1.” If “0” was returned, no new users joined in during the tests. And if “1” was returned, one new user joined in. And we used the same approaches in Section 6.2.1 to generate the test data for the new user.

**Definition 7**

Migration Ratio (mr). In the cloud, mr was defined as the proportion of migrated VMs in all the VMs, which is denoted as (8). Consider

(8) $$\begin{array}{c} \text{mr}=\frac{\eta_{m}}{\eta_{\text{all}}}*100\%, \end{array}$$

where *η*_*m*_ referred to the number of migrated VMs and *η*_all_ referred to the number of all VMs in the cloud.

In this experiment, we designed the following steps. (1) Generating the VMs traces and calculating the ACIR sets. (2) Generating the VMs requests and using SVMS and RVMPS to obtain the placement solutions. (3) Recounting the VMs traces after the VMs placement and recalculating the ACIR sets for all the users. (4) Using the VMs migration solution calculated algorithm to get the migration solutions to guarantee the isolation between conflict users.

**Figure 8:**
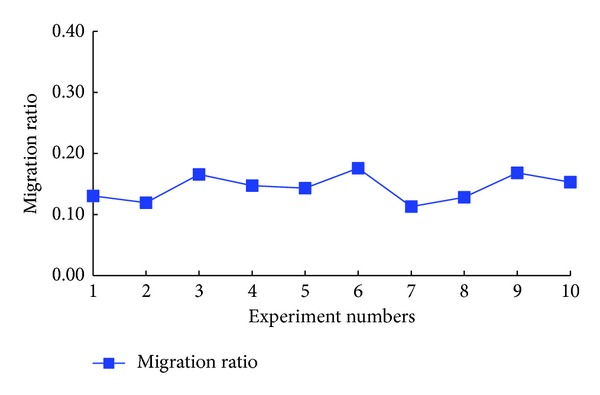
Migration ratios.

**Figure 9:**
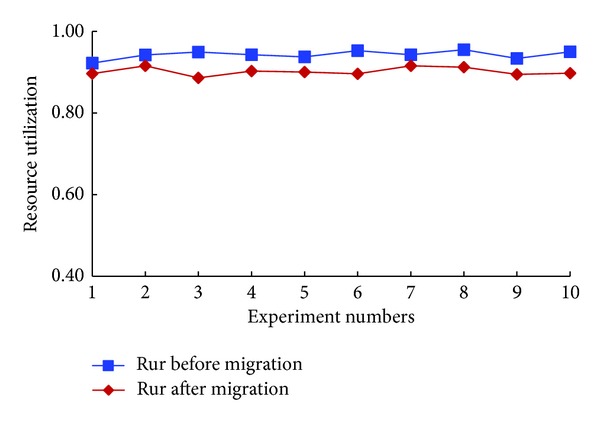
Resource utilization rate.

Finally, we could obtain the experimental results as shown in Figures 8 and 9.

In Figure 8, we demonstrated the results of migration ratios. As shown in this figure, the migration ratios were about 13% in the repeated tests, which meant that we should migrate about 13% VMs to guarantee the isolation. In our opinion, the migration ratios were a bit large. We believed this was mainly because the requested VMs were independent on the history VMs traces. Then the update VMs traces would be very different from the history VMs traces, which resulted in the big difference in the ACIR of users.

We compared the resource utilization rate before and after the VMs migration by using SVMS in Figure 9. As shown in this figure, the resource utilization rate fluctuated around 90% before the migration. While, after migration, rur fluctuated around 86%, which decreased a bit compared with the results before migration. Through this experiment, we could draw the conclusion that to adapt the changed ACIR, more physical resources were needed.

*Brief Summary*. In this section, we mainly focused on evaluating SVMS. First, we considered the isolation and resource utilizations in the case of one placement cycle where the ACIR sets did not change. Then, we considered the case of multiplacement cycles, where the ACIR sets changed. By analyzing the experimental results, we could draw the following conclusions. (1) SVMS could guarantee the isolation between the conflict users effectively. In our repeated tests, the probability of VMs owned by conflict users being located in the same host was 0. (2) The joining in of the new user had no impact on the isolation. However, the resource utilization rate would decrease. (3) To guarantee the isolation after the change of ACIR, we should migrate a portion of VMs and start more hosts.

## 7. Conclusions

We proposed a security VMs management scheme (SVMS) for eliminating the SCA in the cloud. The major insights are that the sophisticated decision making system and specific monitoring systems are not required. In SVMS, we only need the agent system to collect the VMs traces for analyzing the constraint relations, and we can insure that the VMs owned by conflict users are placed to the different hosts. We should notice that SVMS makes a trade-off between the security and resource utilization rates, which means that SVMS needs more resources compared with other resource-awareness placement schemes.

The work in this paper is very preliminary, but demonstrates that it is feasible to reduce the vulnerability by VMs placement and migration. And we believe that there are still some limitations and challenges that remain to be solved. First, in the current solution, we consider that the users should be isolated from each other according to their VMs traces, which is too strict for eliminating the SCA in some ways. So, more efficient approaches for conflict analysis are needed, even though we believe that SVMS has a flexible framework which can adapt to different approaches for calculating the conflict relations of users. Second, according to the experimental results, the cost of VMs migration is large. So, it is necessary to design a more efficient migration approach. We believe the VCG mechanism in game theory would be a good tool for solving this problem. Third, we would like to examine the trade-offs between security (isolation), resource utilization rate, and performance degradation. Therefore, we can propose the security solutions which can meet the individual needs of users. The issues mentioned above are left as future works.

## Figures and Tables

**Algorithm 1:**
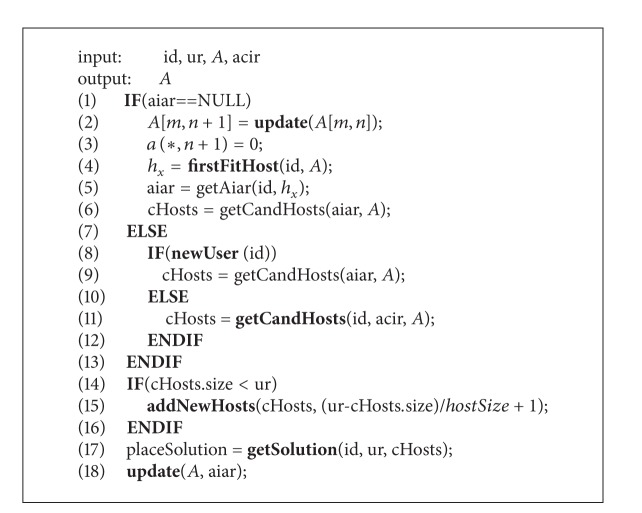
VMs placement solution calculated algorithm.

**Algorithm 2:**
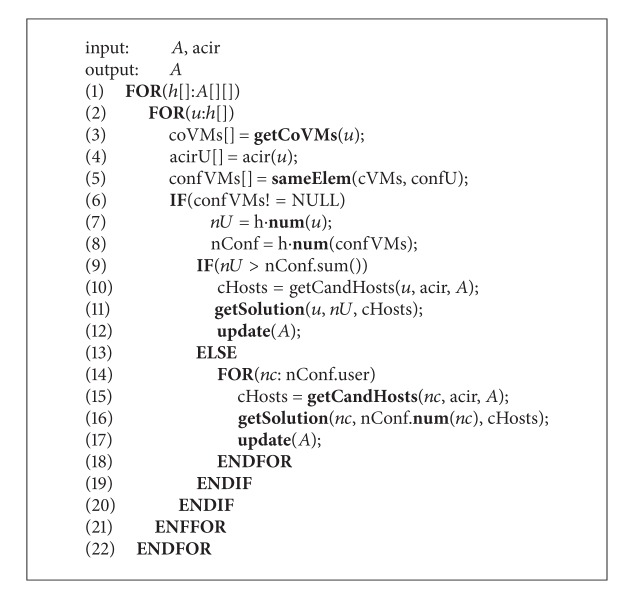
VMs migration solution calculated algorithm.

**Table 1:** Example of information.

ID	User	Time domain	Support
id_*i*_	*u*_*j*_	td_*k*_	*σ*_*l*_

## Appendix

Theorem A.1 The isolation rules meet the *-security property: user *u* can assess host *h*; then no host *h*′ which is accessed by *u* is aggressive conflict with *h*.

Proof We use the method of proof by contradiction. For any two different users, *u*_*j*_ and *u*_*k*_, satisfy the condition that *u*_*k*_∈ ACIR(*u*_*j*_) ∧ *a*(*i*, *j*) = 1∧*R*(*i*, *k*) is granted. In this scenario, *a*(*i*, *k*) may be assigned to “0” or “1.”

1. If *a*(*i*, *k*) = 0, according to Rule 2, when *R*(*i*, *k*) is granted, we can obtain that *a*(*i*, *k*) = 1∧*a*(*i*, *j*) = −1, which means that *R*(*i*, *j*) will be rejected. The conclusion contradicts the condition.
2. If *a*(*i*, *k*) = 1, according to Rule 3, when *R*(*i*, *k*) is granted, we can obtain that *a*(*i*, *k*) = 1∧*a*(*i*, *j*) = −1, which means that *R*(*i*, *j*) will be rejected. The conclusion contradicts the condition.

Combining the two cases, we can conclude that if *u* is granted to access *h*′ which is aggressive conflict with *h*, then *u* cannot access *h*. The conclusion contradicts the condition. So the condition does not hold. So, our proposed isolation rules meet *-security property.

Corollary A.2 The isolation rules meet the simple-security property.

Proof According to the definition of *-security property, if the rules meet *-security property, they should meet simple-security property.
